# Complete Genome Sequence of a Sapporo Virus GV.2 Variant from a 2016 Outbreak of Gastroenteritis in Sweden

**DOI:** 10.1128/genomeA.01446-16

**Published:** 2017-02-02

**Authors:** Björn Hallström, Nina Lagerqvist, Maria Lind-Karlberg, Sofia Helgesson, Per Follin, Maria-Pia Hergens, Joanna Nederby-Öhd, Thomas Tolfvenstam, Erik Alm

**Affiliations:** a Public Health Agency of Sweden, Solna, Sweden; b Stockholm County Council, Stockholm, Sweden; c Karolinska Institutet, Solna, Sweden

## Abstract

During an outbreak of acute gastroenteritis in Sweden when laboratory routine diagnostics failed to detect a causative agent, Sapporo virus was detected in stool specimens using electron microscopy (M.-P. Hergens, J. Nederby Öhd, E. Alm, H. Hervius Askling, S. Helgesson, M. Insulander, N. Lagerkvist, B. Svennungsson, M. Tihane, T. Tolfvenstam, P. Follin, unpublished data). Whole-genome sequencing revealed a Sapporo virus variant clustering with genogroup V.

## GENOME ANNOUNCEMENT

Total RNA was extracted from 200-µL aliquots of two stool supernatants (S3 and S6) using MagDEA Dx SV reagent and the magLEAD instrument (Precision System Science). The RNA was eluted in 50 µL and stored at −80°C pending analysis. A barcoded RNA library was prepared using 10 µL of total RNA and the Ion total RNA-seq kit for the AB library builder system (Thermo Fisher Scientific). The preparation was done in duplicate with different fragmentation times for RNA isolated from S3 and S6. After library construction, the libraries were amplified on a Veriti Thermal Cycler according to the protocol in the manual of the Ion total RNA-seq kit (Thermo Fisher Scientific) with the following modification: a total of 22 cycles for libraries prepared from sample S3 and a total of 16 cycles for libraries prepared from samples S6 were applied for the amplification step. The amplified libraries were size-selected (range between 120 and 292 bp) using a Pippin prep (Sage Science, USA) and then purified using AMPure beads (Beckman Coulter, Inc., USA). The libraries were pooled to a final concentration of 50 pM and clonally amplified using the Ion Chef system and then used for sequencing on an Ion S5 XL instrument using a 540 chip and Ion 540 Kit-Chef (Thermo Fisher Scientific).

The sequence data was assembled using CLC assembly cell (version 4.4.2). For sample S3, three contigs originating from SaV were detected through a BLAST search to a reference genome (GenBank accession no. DQ366344). The three contigs, which contained large and exact overlaps with each other, were merged to a single sequence covering the whole genome, with a total size of 7,531 nucleotides. The data from samples S3 and S6 were mapped to the assembled genome sequence using BWA 0.7.2 (1) to correct for assembly errors, quantify the viral content, and detect differences between the two samples. Genome sequences were matched against the NCBI nucleotide collection using BLASTn with default settings to determine similarity to earlier sequenced isolates.

Phylogenetic analysis of the capsid gene shows that the sequences of S3 and S6 cluster with GV but are clearly separated from almost all other isolates in the genogroup. The sequences of S3 and S6 form a distant clade together with sequences originating from Japan and Italy (AB433783, AB473784, and JQ303049). The closest published complete genome was published in 2015 from an outbreak in Japan, GenBank accession no. AB775659 (2).

### Accession number(s).

This whole-genome shotgun project has been deposited in GenBank under the accession no. KY040366. The version described in this paper is the first version.
